# Antigen-dependent and –independent contributions to primary memory CD8 T cell activation and protection following infection

**DOI:** 10.1038/srep18022

**Published:** 2015-12-10

**Authors:** Matthew D. Martin, Vladimir P. Badovinac

**Affiliations:** 1 Interdisciplinary Program in Immunology, University of Iowa Carver College of Medicine, University of Iowa, Iowa City, Iowa, USA; 2 Department of Pathology, University of Iowa Carver College of Medicine, University of Iowa, Iowa City, Iowa, USA

## Abstract

Memory CD8 T-cell activation, including expression of IFN-γ and granzymeB, can be induced by antigen (Ag)-dependent signals through the T-cell-receptor, or by pathogen-derived inflammatory cytokines in an Ag-independent manner. Recent studies have come to conflicting results regarding the contributions of Ag and/or inflammation to memory CD8 T-cell activation. Additionally, research has indicated that inflammation-driven CD8 T-cell responses during un-related infections (bystander activation) have the potential to provide protection, but whether protection occurs in immuno-competent hosts is unclear. To investigate these questions, we examined activation of virus-specific memory CD8 T-cells following infection with *L. monocytogenes* either expressing or not cognate Ag. We show that Ag and inflammation act synergistically *in vitro* to induce memory activation. *In vivo*, we found that when memory CD8 T-cells significantly contribute to clearance of infection, early activation and continued responses by these cells are enhanced by cognate Ag recognition. Mechanistically, we show that bystander responses by memory are dependent upon the dose of infection and the amount of inflammation elicited following infection and are able to provide protection in IFN-γ deficient mice, but not in immuno-competent hosts. The data elucidate the requirements for memory CD8 T-cell activation and the protective role of bystander responses.

Following infection, memory CD8 T cells become activated and produce effector molecules providing immune hosts with enhanced protection against invading microorganisms^1^. Therefore, understanding the requirements for memory CD8 T cell re-activation may aid in the design of protective vaccines. It is well established that memory CD8 T cell production of cytokines and lytic molecules is induced downstream of TCR signaling upon cognate Ag recognition. However, memory CD8 T cells can be activated and produce IFN-γ and granzymeB (GrB) in a cytokine dependent, Ag-independent manner^2^,^3^,^4^,^5^,^6^. Therefore, activation following infection with pathogens expressing Ags recognized by memory CD8 T cells could be driven by either cognate Ag recognition or by inflammatory cytokines. Recent studies have come to differing conclusions as to the relative importance of cognate Ag recognition for eliciting memory CD8 T cell functions. One recent report indicated that early activation of circulating memory CD8 T cells occurs independently of cognate Ag recognition^7^. However, a different study indicated that the ability of tissue resident memory CD8 T cells to sense infection resulting in the production of IFN-γ, local chemokine production, and the recruitment of immune cells, requires cognate Ag recognition^8^. While these conclusions appear to be contradictory, they both could be true if the influence of cognate Ag on memory CD8 T cell activation is context dependent. However, these studies indicate that the contribution of Ag-dependent and independent signals to memory CD8 T cell reactivation has yet to be clearly defined.

Cytokine production and targeted killing of infected cells promote clearance of pathogens recognized by memory CD8 T cells following infection. In the same manner, effector cytokines and lytic molecules produced by memory CD8 T cells activated in an Ag- independent manner (bystander activation) could provide protection against non-related infections. Bystander responses have been shown to provide protection against infection with *L. monocytogenes* (LM) or *S. typhimurium* in IFN-γ deficient or NK cell depleted hosts^4^,^9^,^10^,^11^, suggesting that bystander responses could represent an important protective immune response upon infection with diverse pathogens. However, experiments examining the protective role of bystander CD8 T cell responses in immuno-competent hosts have yielded conflicting results. One report has indicated that bystander responses following bacterial infection are protective^7^, while another report has indicated that they provide little to no protection^12^. Additionally, multiple studies examining viral infection have indicated that only memory CD8 T cells that recognize Ag due to TCR cross-reactivity are able to provide protection against infection with unrelated viruses^13^. Therefore, it is unclear if bystander responses by memory CD8 T cells provide protection in immuno-competent hosts.

In this study we address the contribution of Ag and inflammation to memory CD8 T cell activation, and protection provided by virus-specific bystander memory CD8 T cells following LM infection. We show that Ag and inflammatory cytokines synergize *in vitro* to induce memory CD8 T cell activation. *In vivo*, responses by memory CD8 T cells that significantly contributed to clearance of infection were enhanced upon infection with pathogens expressing cognate Ag. Memory CD8 T cell bystander responses during unrelated infection were dependent upon the dose of infection and the amount and duration of inflammation elicited following infection, and in agreement with previous literature, we show that bystander IFN-γ production by memory CD8 T cells during un-related infection with LM is protective in IFN-γ deficient hosts. However, bystander memory CD8 T cell responses in immuno-competent hosts provided no protection. Our findings elucidate the role of Ag in memory CD8 T cell activation and protection provided by bystander memory CD8 T cell responses following non-related bacterial infections.

## Results

### Ag and inflammation act synergistically *in vitro* to induce memory CD8 T cell activation

**Figure 1:**
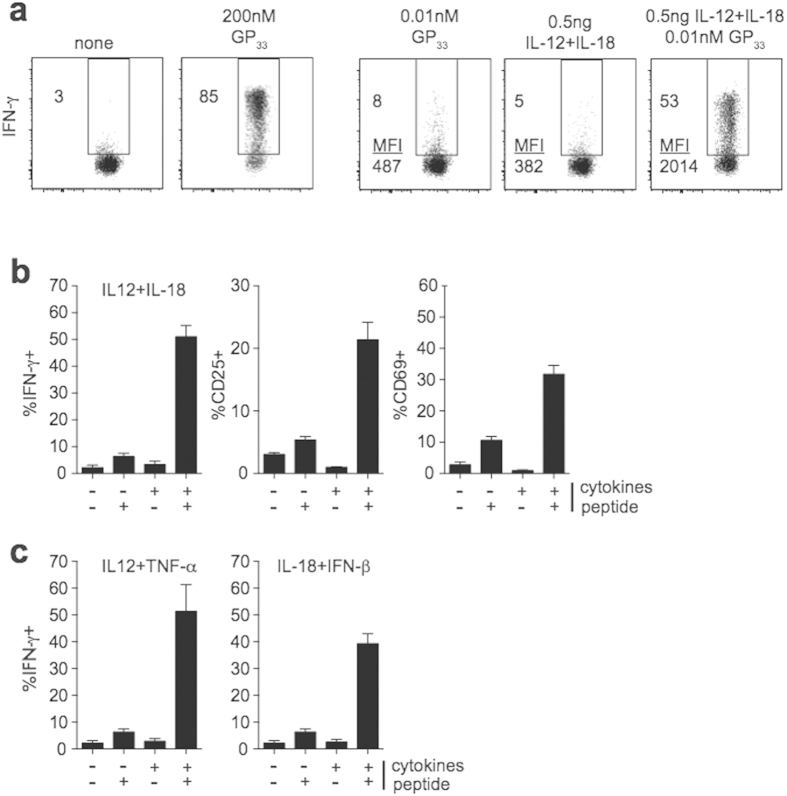
Ag and inflammation act synergistically *in vitro* to induce memory CD8 T cell activation. (**a**) Representative dot plot showing IFN-γ production by P14 cells incubated for 5 hrs in the presence of the indicated concentrations of GP_33_ peptide and/or the indicated concentrations of rIL-12 and IL-18. (**b**) Percentages of P14 cells producing IFN-γ after 5 hour incubation in the presence (+) or absence (−) of GP_33_ peptide (0.01 nM) and/or rIL-12 and IL-18 (0.5 ng each) or (**c**) IL-12 and TNF-α or IL-18 and IFN-β. Data shown are the mean +SEM of one representative experiment out of greater than three independent experiments with three mice per group.

To determine how Ag and inflammation might interact to influence memory CD8 T cell activation during infection, we devised an *in vitro* system that allowed us to examine their effects on memory CD8 T cell activation separately, or in combination. At the very onset of infection, Ag and inflammation are present at low levels. We therefore incubated memory P14 cells with low concentrations of inflammatory cytokines that elicit activation of memory CD8 T cells^2^,^3^,^4^,^5^,^6^,^14^, low concentrations of cognate Ag, or a combination of cytokines and Ag. Less than 10% of memory CD8 T cells that were capable of responding to Ag (Fig. 1a left panels) became activated following incubation with low concentrations of GP_33_ peptide or recombinant (r)IL-12 and IL-18 alone (Fig. 1a,b). However, a large percentage of memory CD8 T cells produced IFN-γ and expressed the activation markers CD25 and CD69 when incubated with low levels of GP_33_ peptide and rIL-12 and IL-18 (Fig. 1a,b), or rIL-12 and TNF-α or rIL-18 and IFN-β (Fig. 1c). These data suggest that Ag and inflammation have the capacity to synergize to induce CD8 T cell activation, and that low levels of Ag and inflammation present at the onset of infection may lead to enhanced CD8 T cell responses.

### Early activation of memory CD8 T cells that do not significantly contribute to clearance of infection is not influenced by cognate Ag

**Figure 2:**
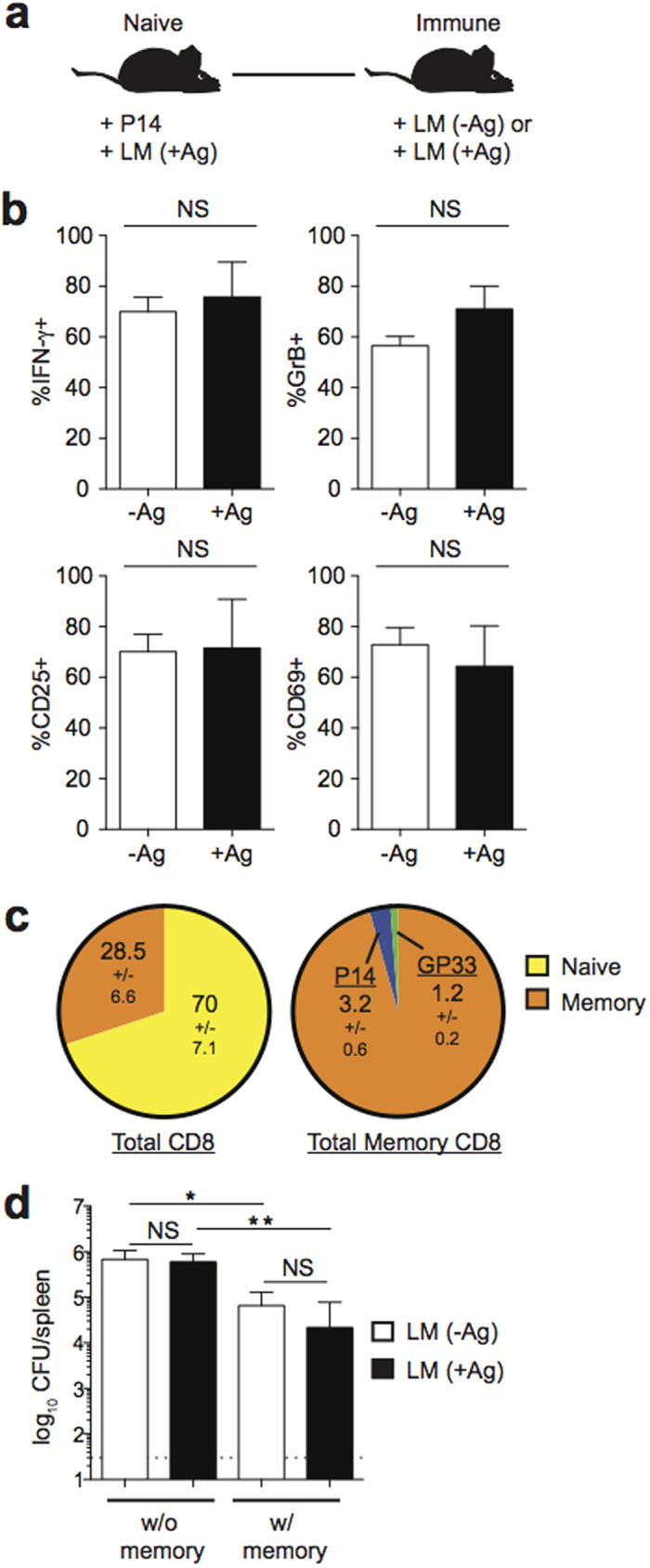
Cognate Ag does not influence early activation of memory CD8 T cells that do not significantly contribute to clearance of infection. (**a**) Experimental design. Following adoptive transfer of naïve P14 cells, mice were infected with 1 × 10^4^ CFU Vir LM-GP_33_. Mice were given a 2° infection with 1 × 10^6^ CFU Vir LM (LM −Ag) or Vir LM-GP_33_ (LM +Ag) >30 days later. (**b**) Percentage of P14 cells producing IFN-γ or granzymeB, or expressing CD25 or CD69 8 hrs post 2° infection. (**c**) Percentage of memory CD8 T cells out of total CD8 T cells (left) and P14, GP_33_ tetramer, or LM-specific memory CD8 T cells out of total memory CD8 T cells (right) prior to 2° infection. (**d**) Bacterial CFUs in the spleen 8 hrs post 1° or 2° infection. Data shown are the mean +SEM of one representative experiment out of two independent experiments with three mice per group. NS, not significant. *p < 0.05. **p < 0.01.

Our *in vitro* findings suggested that cognate Ag might enhance memory CD8 T cell responses during re-infection. In contrast, a recent study by Soudja *et al.* concluded that early activation of memory CD8 T cells is not influenced by the presence of cognate Ag^7^. In order to confirm these findings and to attempt to explain why cognate Ag does not influence early activation of memory CD8 T cells using a system similar to that used by Soudja *et al.*, we generated memory P14 cells by adoptively transferring small numbers of naïve P14 cells into naïve B6 mice that were then infected with LM expressing the GP_33_ epitope. At a memory time point, mice were given a 2° infection with LM either expressing or not expressing GP_33_ (Fig. 2a). In this system, P14 cell memory responses occur in the presence of LM-specific memory responses and in the context of an infection either expressing cognate Ag (LM+Ag), or in the absence of cognate Ag (LM−Ag). Similarly to Soudja *et al.*, we found that the percentage of cells producing IFN-γ or GrB, or expressing the activation markers CD25 or CD69 was similar regardless of the presence of cognate Ag (Fig. 2b). However, in this system, memory P14 cells constituted a minor fraction of the total pathogen-specific response (Fig. 2c). Additionally, while prior infection with LM led to a reduction in bacterial burdens, responses by P14 cells did not significantly enhance LM clearance beyond that mediated by the large LM-specific memory CD8 T cell population (Fig. 2d). These data suggest that cognate Ag recognition is not required for activation and IFN-γ production by memory CD8 T cells that do not significantly contribute to clearance of infection.

### Cognate Ag and continued infection drive early activation and sustained responses of memory CD8 T cells that contribute to pathogen clearance

**Figure 3:**
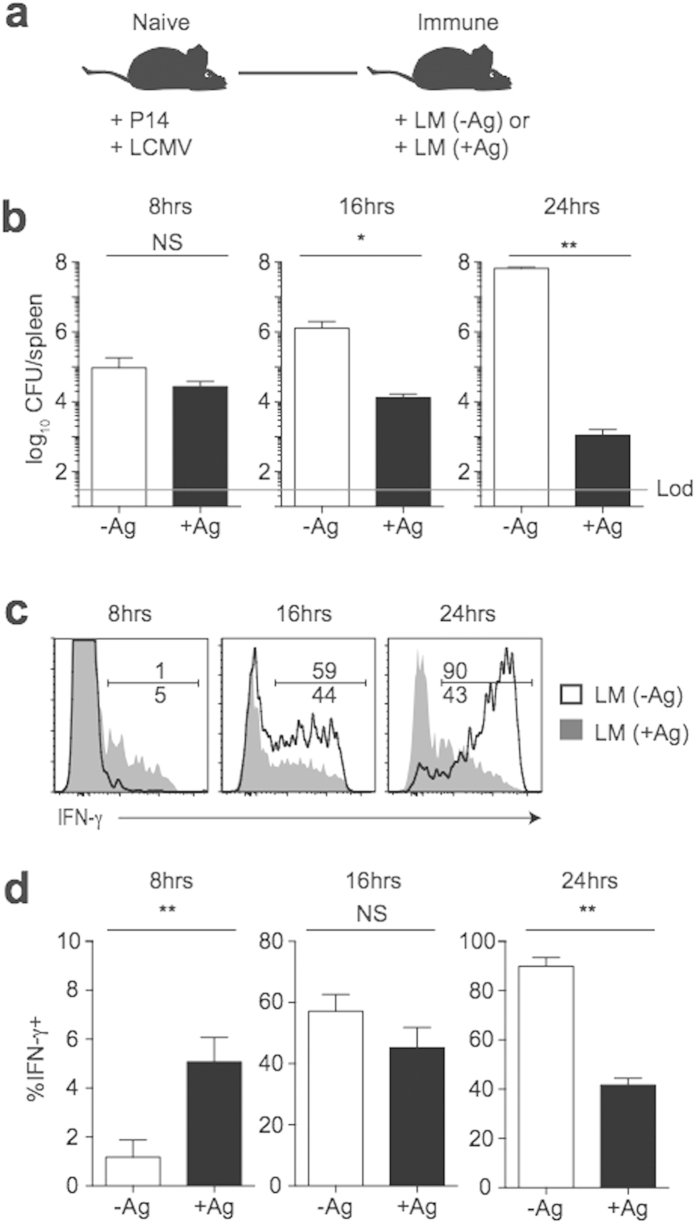
Ag and continued infection promote early activation and prolonged responses by memory CD8 T cells that contribute to clearance of infection. (**a**) Experimental design. Following adoptive transfer of naïve P14 cells, mice were infected with LCMV (2 × 10^5^ PFU). Mice were given a 2° infection with 1 × 10^5^ CFU Vir LM (LM −Ag) or Vir LM-GP_33_ (LM +Ag) >30 days later. (**b**) Bacterial CFUs in the spleen at the indicated times post 2° infection. (**c**) Representative histograms of IFN-γ production by P14 cells infected with Vir LM (LM (-Ag)- clear histograms) and Vir LM-GP_33_ (LM (+Ag)- grey histograms) at the indicated times post 2° infection. (**d**) Percentage of P14 cells producing IFN-γ at the indicated times post 2° infection. Data shown are the mean +SEM of one representative experiment out of three independent experiments with three mice per group. NS, not significant. *p < 0.05. **p < 0.01.

In contrast to bacterial and/or viral infection which elicits a large polyclonal memory population that is capable of responding and contributing to pathogen clearance during re-infection, subunit vaccination results in the generation of memory CD8 T cells recognizing one or a small number of Ags present in invading microbes. To examine how Ag and inflammation influence the activation of a population of memory CD8 T cells specific for a single Ag that respond in the absence of a large polyclonal memory response, we generated memory P14 cells following infection with LCMV. At a memory time point, mice were given a 2° infection with virulent (Vir) LM either expressing or not expressing GP_33_ (Fig. 3a). In this system, P14 cells and endogenous GP_33_-specific cells are the only Ag-specific memory cells present, and P14 cell responses occur in the context of an infection either expressing (LM+Ag) or not expressing (LM−Ag) cognate Ag. Clearance of LM was mediated by memory P14 cells, as mice infected with LM expressing GP_33_ displayed significantly lower CFUs in the spleen 16 and 24 h post infection (Fig. 3b). Importantly, IFN-γ production by memory P14 cells occurred faster in response to LM expressing GP_33_, and responses waned as infection was cleared (Fig. 3c,d). Importantly, bystander activation of memory P14 CD8 T cells was truly driven by the infection since those cells did not show an increase in production of IFN-γ and GrB, and/or an increase in the expression of CD25 and CD69 when analyzed in non-infected, LCMV immune mice (Fig. S1). These data suggest that early activation of memory CD8 T cells is enhanced by cognate Ag recognition, and that continued responses by memory CD8 T cells are dependent upon continued infection.

**Figure 4:**
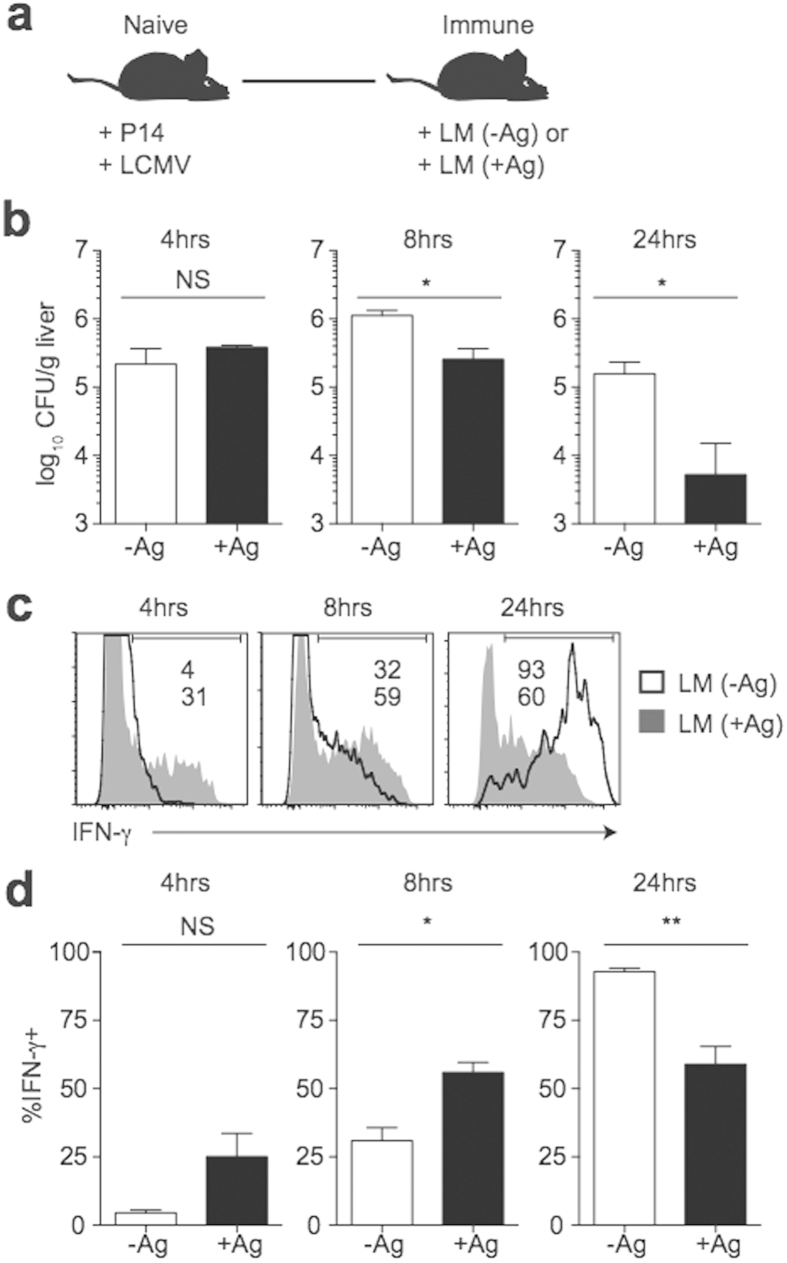
Ag influences early activation of memory CD8 T cells that contribute to clearance of infection regardless of LM virulence. (**a**) Experimental design. Following adoptive transfer of naïve P14 cells, mice were infected with LCMV (2 × 10^5^ PFU). Mice were given a 2° infection with 5 × 10^6^ CFU Att LM (LM −Ag) or Att LM-GP_33_ (LM +Ag) >30 days later. (**b**) Bacterial CFUs in the spleen at the indicated times post 2° infection. (**c**) Representative histograms of IFN-γ production by P14 cells infected with Att LM (LM (-Ag)- clear histograms) and Att LM-GP_33_ (LM (+Ag)- grey histograms) at the indicated times post 2° infection. (**d**) Percentage of P14 cells producing IFN-γ at the indicated times post 2° infection. Data shown are the mean +SEM of one representative experiment out of two independent experiments with three mice per group. NS, not significant. *p < 0.05. **p < 0.01.

During the initial stages of infection with Vir LM, bacterial levels are low, resulting in low levels of bacterial Ag^15^,^16^. While memory CD8 T cell responses were significantly enhanced early following infection with Vir LM expressing GP_33_ compared to infection not expressing GP_33_, only a small percentage of memory CD8 T cells were activated at early time points (Fig. 3c,d). In contrast to Vir LM infection, CD8 T cell responses peak earlier following infection with attenuated^9^ *actA* deficient LM, and initial levels of bacteria and Ag are higher^15^,^17^,^18^,^19^. In order to examine the effects of Ag and inflammation on early memory CD8 T cell responses during an infection where levels of Ag are abundant, we generated memory P14 cells following LCMV infection and at a memory time point infected mice with Att LM either expressing or not expressing GP_33_ (Fig. 4a). While levels of bacteria were similar early after Att LM infection (Fig. 4b), a greater percentage of memory CD8 T cells responding in the presence of cognate were activated at early time points, and responses waned as infection was cleared (Fig. 4c,d). Taken together, these data suggest that early activation of memory CD8 T cells is enhanced by cognate Ag recognition.

### Bystander memory CD8 T cell responses are influenced by dose of infection and amount of inflammation elicited upon infection

**Figure 5:**
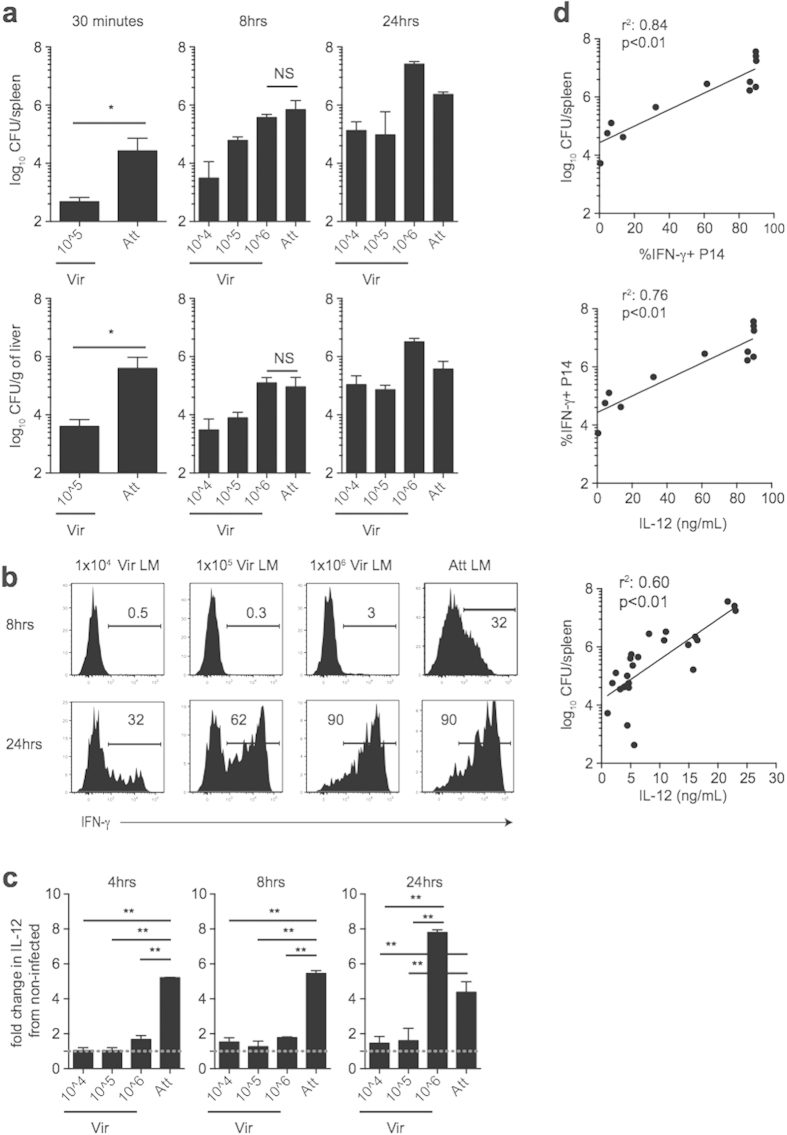
Magnitude of bystander memory CD8 T cell responses correlates with the level of infection and inflammation elicited upon infection. (**a-d**) Mice containing memory P14 cells generated following LCMV infection (2 × 10^5^ PFU) were given a 2° infection with Att LM (5 × 10^6^ CFU) or Vir LM (1 × 10^4^, 1 × 10^5^ or 1 × 10^6^ CFU) not expressing GP_33_ (LM −Ag) >30 days later. (**a**) Bacterial CFUs in the spleens and livers following the indicated 2° infections at the indicated times post infection. (**b**) Representative histograms of IFN-γ production by P14 cells at the indicated times post 2° infection. (**c**) Fold increase in the level of serum IL-12 following the indicated 2° infections at the indicated times post infection relative to mice not receiving 2° infection (dashed line). (**d**) CFUs of bacteria recovered from the spleens of mice plotted vs. the percentage of IFN-γ+ P14 cells 24 hrs after infection (upper top), percentage of IFN-γ+ P14 cells plotted vs. the concentration of serum IL-12 24 hrs after infection (middle), and CFUs of bacteria recovered from the spleens of mice plotted vs. the concentration of serum IL-12 8 and 24 hrs after infection (bottom). Data shown are the mean +SEM of one experiment with three to four mice per group. *p < 0.05 **p < 0.01.

We noticed that early (8 hours (hrs) post infection) bystander activation of memory CD8 T cells was enhanced following Att LM infection (Fig. 4c,d) compared to Vir LM infection (Fig. 3c,d). Because the dose of infection differed in the two experiments, we reasoned that the magnitude of bystander memory CD8 T cell responses might be dependent upon the dose of infection. To test this, we infected LCMV-immune mice containing memory P14 cells with Att LM (5 × 10^6^ CFU) or with titrating doses of Vir LM (1 × 10^4^–1 × 10^6^ CFU). 30 minutes following infection, the recoverable CFUs from the spleens and livers of mice infected with 5 × 10^6^ CFU Att LM was significantly higher than that of mice infected with 1 × 10^5^ CFU Vir LM (Fig. 5a). Corresponding to the higher initial dose, bystander IFN-γ production was detectable in mice infected with Att LM by 8 hrs post infection while it was not detectable in mice infected with 1 × 10^4^ or 1 × 10^5^ CFU Vir LM (Fig. 5b). By 24 hrs post infection when bystander responses were detectable in mice infected with Vir LM, the percentage of memory P14 cells producing IFN-γ in a bystander fashion increased with increasing dose of infection (Fig. 5b) suggesting that dose of inoculum determines, at least in part, the bystander activation of memory CD8 T cells.

However, we also noted that while the level of infection was not significantly different 8 hrs post infection between mice that were infected with 1 × 10^6^ CFU Vir LM, and 5 × 10^6^ Att LM (Fig. 5a), the percentage of bystander activated P14 cells was higher in mice infected with Att LM (Fig. 5b). Because inflammatory cytokines have been shown to drive bystander activation of memory CD8 T cells *in vitro*^2^,^4^, we reasoned that inflammation (the amount and duration) elicited following infection might be a determining factor that controls bystander responses of memory CD8 T cells *in vivo*. When we examined the systemic levels of IL-12 in the serum of infected mice in relation to mice that did not receive a 2° LM infection, levels of IL-12 were significantly greater in mice that received Att LM infection than mice that received any dose of Vir LM infection at 4 and 8 hrs post infection (Fig. 5c). By 24 hrs post infection when bystander IFN-γ production by memory CD8 T cells was highest in mice that received 5 × 10^6^ CFU Att LM and 1 × 10^6^ CFU Vir LM infection (Fig. 5b), serum IL-12 levels were significantly greater in mice that received either of these two infections compared to mice that received 1 × 10^4^ or 1 × 10^5^ CFU Vir LM infection (Fig. 5c). When linear regression analysis was used to examine the correlations between the dose of infection, the percentage of P14 cells producing IFN-γ in a bystander manner, and the level of systemic IL-12, statistically significant correlations were observed between **I)** the CFUs of bacteria recovered from the spleens 24 hrs post infection and the percentage of P14 cells producing IFN-γ; **II)** the percentage of P14 cells producing IFN-γ and the levels of serum IL-12, and **III)** the CFUs of bacteria recovered from the spleens 8 and 24 hrs post infection and the levels of serum IL-12 (Fig. 5d). Taken together, these data indicate that bystander responses by memory CD8 T cells are influenced by the dose of infection and the amount and duration of systemic inflammation elicited upon infection.

### Bystander memory CD8 T cell responses provide protection in IFN-γ deficient, but not immuno-competent hosts

**Figure 6:**
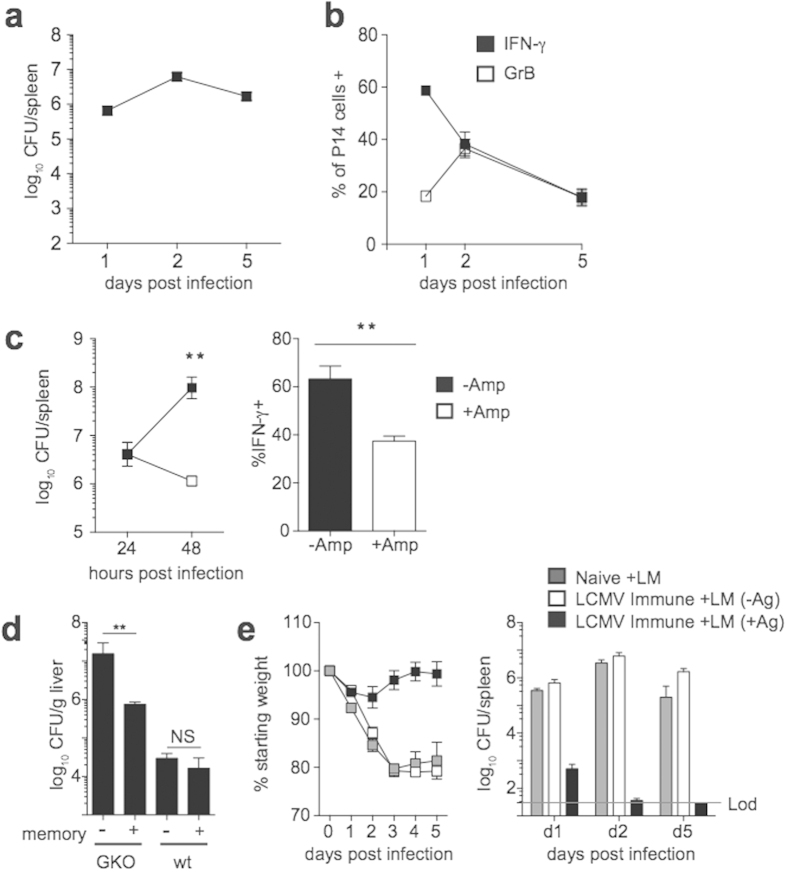
Bystander responses by memory CD8 T cells provide protection against LM in IFN-γ deficient, but not IFN-γ sufficient hosts. (**a,b**) Following adoptive transfer of naïve P14 cells, mice were infected with LCMV (2 × 10^5^ PFU). Mice were given a 2° infection with 1 × 10^4^ CFU Vir LM (LM −Ag) >30 days later. (**a**) Bacterial CFUs in the spleen of LCMV immune mice at the indicated day post 2° infection. (**b**) Percentage of P14 cells producing IFN-γ or GrB at the indicated day post 2° infection. (**c**) B6 mice received adoptive transfer of naïve P14 cells, and mice were infected with LCMV (2 × 10^5^ PFU). Mice were given a 2° infection with 1 × 10^6^ CFU Vir LM (LM −Ag) >30 days later. A group of mice were treated with ampicillin 24 hrs post 2° infection. Left, Bacterial CFUs in the spleen at the indicated times post 2° infection, and right, percentage of P14 cells producing IFN-γ 48 hrs post 2° infection. (**d**) 4 × 10^5^ memory P14 cells were transferred (+) or not (−) into IFN-γ deficient or normal B6 (wt) mice, and mice were infected with 1 × 10^4^ CFU Vir LM. Bacterial CFUs in the liver 2 days following infection. (**e**) Following adoptive transfer of naïve P14 cells, mice were infected with LCMV (2 × 10^5^ PFU). Mice were given a 2° infection with 1 × 10^4^ CFU Vir LM (LM −Ag) or Vir LM-GP_33_ (LM +Ag) >30 days later. Naïve mice received no adoptive transfer of P14 cells or LCMV infection. Morbidity (left) and bacterial CFUs in the spleen (right) at the indicated day post LM infection (1 × 10^4^ CFU Vir LM or Vir LM-GP_33_). Data shown are the mean +SEM of one representative experiment out of two independent experiments with three to five mice per group.**p < 0.01.

Non-Ag driven bystander responses by memory CD8 T cells persisted up to 24 hrs following LM infection (Figs 3 – 5). To examine if bystander responses continue beyond 24 hrs, IFN-γ and GrB production by P14 cells in LCMV immune mice was examined from 1–5 days after infection with Vir LM not expressing GP_33_. Infection persisted up to 5 days following infection, (Fig. 6a) and IFN-γ and GrB producing bystander activated P14 cells could be detected at all time points examined (Fig. 6b). However, the percentage of IFN-γ producing P14 cells decreased from day 1 to d5. Bystander responses were dependent on the level of infection, as reducing the level of infection through antibiotic treatment resulted in a decreased percentage of P14 cells producing IFN-γ (Fig. 6c). Thus, bystander CD8 T cell responses persist for several days but wane following un-related infections, and responses depend on the level of infection.

To examine if bystander responses by memory CD8 T cells are capable of providing protection during infection with unrelated pathogens, memory P14 cells were either transferred or not transferred into normal B6 or IFN-γ deficient mice that were infected with LM not expressing GP_33_. As has been reported^20^,^21^, IFN-γ is important for clearance of primary LM infection, as bacterial burdens were 2-3 logs higher in the livers of IFN-γ knockout^5^ mice compared to B6 mice 2 days following infection (Fig. 6d). Bacterial colony forming units (CFUs) were significantly reduced in GKO mice that received transferred memory P14 cells indicating that in the absence of normal IFN-γ production, bystander CD8 T cells provide protection against unrelated infection. However, bacterial CFUs were not significantly different between B6 mice that received or did not receive transferred memory P14 cells (Fig. 6d), indicating that bystander CD8 T cell responses provide minimal protection in mice with a full complement of IFN-γ producing cells.

Following infection, the number of memory CD8 T cells present in an intact mouse is greater than that present following adoptive transfer. To determine if bystander responses by a large population of memory CD8 T cells are protective against non-related infection with LM, naïve or LCMV immune mice were infected with LM expressing or not expressing GP_33_. An approximately 1LD_50_ dose of LM (1 × 10^4^ CFU) was used in order to enable survival and analysis over an extended period of time. Ag-directed memory CD8 T cell responses provided protection, as LM-GP_33_ infected mice lost less weight and cleared infection faster than either naïve mice or LCMV immune mice that were infected with LM not expressing GP_33_ (Fig. 6e). However, bystander memory CD8 T cell responses provided no significant protection, as naïve and LCMV-immune mice infected with LM not expressing GP_33_ experienced similar weight loss and displayed similar bacterial burdens in the spleens 1–5 days post infection.

**Figure 7:**
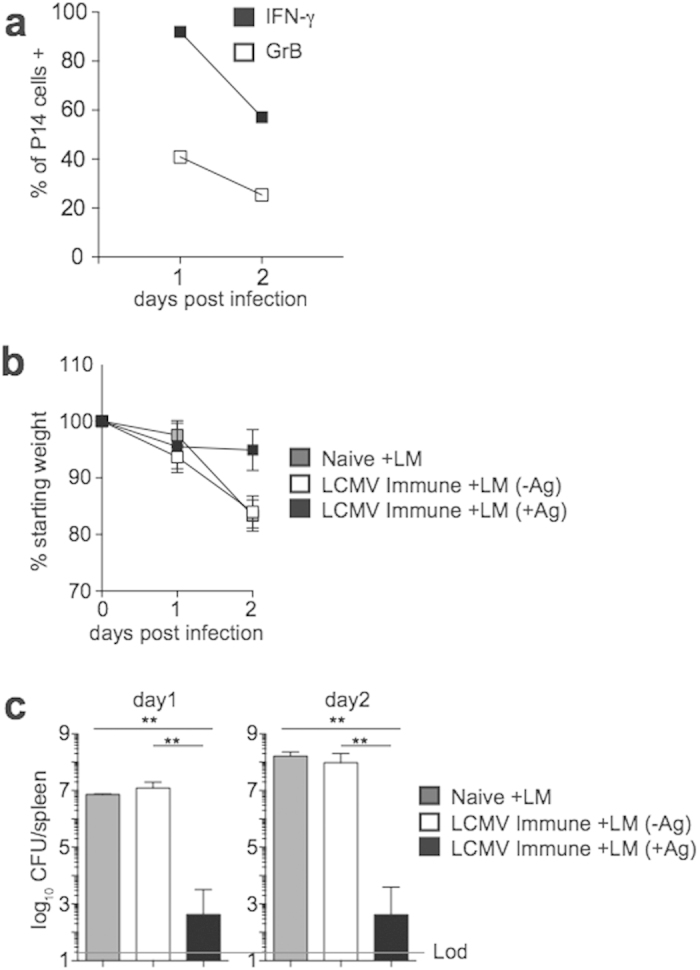
Bystander memory CD8 T cell responses fail to provide protection against high-dose LM infection. (**a**) Following adoptive transfer of naïve P14 cells, mice were infected with LCMV (2 × 10^5^ PFU). Mice were given a 2° infection with 1 × 10^6^ CFU Vir LM (LM −Ag) >30 days later. Percentage of P14 cells producing IFN-γ (black squares) or GrB (open squares) at the indicated day post 2° infection with Vir LM (1 × 10^6^ CFU). (**b**,**c**) Following adoptive transfer of naïve P14 cells, mice were infected with LCMV (2 × 10^5^ PFU). Mice were given a 2° infection with 1 × 10^6^ CFU Vir LM (LM−Ag) or Vir LM-GP_33_ (LM +Ag) >30 days later. Naïve mice received no adoptive transfer of P14 cells or LCMV infection. Morbidity (**b**) and bacterial CFUs in the spleen (**c**) at the indicated day post LM infection. Data shown are the mean +SEM of one representative experiment out of two independent experiments with three mice per group.**p < 0.01.

IFN-γ production by bystander activated CD8 T cells may not provide any additional benefit beyond IFN-γ produced by other cells of the immune system following non-lethal infection. In the case of a lethal infection, however, it is possible that IFN-γ in addition to that produced by other cell types could provide a survival advantage. To examine if bystander responses by memory CD8 T cells provide any protective benefit during a lethal infection, naïve mice or mice containing LCMV-specific memory CD8 T cells were infected with a high dose LM infection (~100 LD_50_). As with low dose infection, a high percentage of bystander activated memory CD8 T cells were present 1 day following high dose LM infection, and responses persisted but waned after day 1 (Fig. 7a). However, while Ag-directed memory CD8 T cell responses protected mice from weight loss and bacterial growth in the spleen, bystander memory CD8 T cell responses did not protect against weight loss or bacterial growth compared to naïve mice (Fig. 7b,c). Taken together, these data indicate that while bystander activated memory CD8 T cells are able to provide protection in hosts lacking a fully functional immune system, bystander responses provide no significant protection during non-related systemic infection with LM in immuno-competent hosts.

## Discussion

We have shown that early activation of memory CD8 T cells is enhanced by cognate Ag. When analyzing 2° memory CD8 T cell activation following homologous infection with LM either expressing or not expressing cognate Ag using a model similar to that described in recent reports^7^, we found that early activation of memory CD8 T cells responding in the presence of a large polyclonal CD8 T cell response was similar regardless of cognate Ag expression by the invading pathogen. However, we demonstrated that in this model the Ag-specific memory cells for which activation was examined did not significantly contribute to clearance of infection, suggesting that activation was primarily driven by inflammation rather than cognate Ag. Conversely, using a heterologous infection model where Ag-specific memory CD8 T cells responded in the absence of other responding memory populations and significantly contributed to pathogen clearance, memory CD8 T cells responded faster in response to pathogens expressing cognate Ag, and responses waned as the infection was cleared. These data are in agreement with our *in vitro* data that showed a synergistic effect of Ag and inflammation for memory CD8 T cell activation and suggest that early activation of memory CD8 T cells responding to pathogens expressing cognate Ag is likely enhanced *in vivo* by a synergistic effect of low levels of inflammation and Ag.

We also showed that bystander activation of memory CD8 T cells is influenced by the infection dose and the level of inflammation elicited following infection. This suggests that the ability of memory CD8 T cells to sense inflammation will impact their ability to become activated early following infection with a pathogen expressing cognate Ag. What parameters, then, might influence the ability of memory CD8 T cells to sense inflammation? Exhausted CD8 T cells have been shown to downregulate the IL-18 receptor and to become unresponsive to unrelated infections^22^. Thus, the nature of the initial infection may influence the ability of memory CD8 T cells to sense inflammation and to become activated following re-infection. Additionally, we have shown that the phenotype, function, and transcriptome, including expression of genes coding for cytokine receptors, of memory CD8 T cells is altered in a stepwise fashion with each additional Ag encounter^23^. Thus, activation of memory CD8 T cells in response to Ag and inflammation may be influenced by Ag stimulation history. Furthermore, the phenotype and function of memory CD8 T cells have been shown to change with time after infection^24^,^25^,^26^,^27^. Our unpublished microarray data indicates that the expression of genes coding for cytokine receptors including components of the IL-12 and IL-18 receptors decreases with time after infection. Therefore, time following Ag encounter may also impact the ability of memory CD8 T cells to sense inflammation and to become activated following infection with a pathogen that expresses cognate Ag.

Non Ag-specific, inflammation driven bystander responses during unrelated infections could provide the host with a protective benefit. Memory CD8 T cells specific for unrelated viruses become activated in humans following HIV^28^,^29^ or Epstein-Barr virus infections^30^. Therefore, determining whether bystander CD8 T cell responses play a protective role during unrelated infections could be of significance to the human population. We have shown that pre-existing virus-specific memory CD8 T cells respond in a bystander manner but provide no significant protection following unrelated LM infection. However, LM results in acute infection characterized by systemic inflammatory cytokine responses^31^, and it is possible that cytokines produced by bystander activated CD8 T cells do not significantly add to systemic levels of cytokines produced by innate cells during the early response to LM. In contrast to systemic infections, immune responses to localized infections are initiated by a smaller number of cells that survey peripheral tissues. Ag-driven responses by resident memory CD8 T cells in barrier tissues provide enhanced protection to localized infections^32^,^33^,^34^,^35^,^36^, but it is unclear if tissue resident memory CD8 T cells can be activated following unrelated infections. This is an important question, as bystander responses by memory CD8 T cells in areas of the body where levels of inflammatory cytokines are low may provide the host with a protective benefit following infection with unrelated pathogens.

In summary, our data elucidate the factors influencing activation of memory CD8 T cells and the protective role of bystander memory CD8 T cells. The data indicate that memory CD8 T cell reactivation is enhanced by Ag and inflammation, and that bystander memory CD8 T cell responses to unrelated systemic bacterial infections are influenced by infection dose and amount of inflammation elicited, but are not protective in immuno-competent hosts. These data have implications for the development of vaccination strategies designed to generate protective memory CD8 T cells against diverse pathogens.

## Methods

### Mice

C57BL/6 (B6), IFN-γ knockout, and P14 mice were bred and maintained in the animal facilities at the University of Iowa at the appropriate biosafety levels. All animal studies were approved by the University of Iowa Institutional Animal Care and Use Committee and met stipulations of the *Guide for the Care and Use of Laboratory Animals* (National Institutes of Health). All animal studies were carried out in accordance with these approved guidelines.

### Bacterial and Viral Infections, Memory Generation, Adoptive Transfers, and Ampicillin Treatment

The Armstrong strain of LCMV and attenuated *actA*- deficient (Att LM) strain DP-L1942 or virulent *Listeria monocytogenes* (Vir LM) strain 1043S expressing and not expressing the GP_33_ epitope were grown and quantified as previously described^24^,^37^. 1° memory P14 cells were generated by adoptively transferring 5 × 10^3^ P14 cells obtained from peripheral blood of naïve P14 mice (Thy1.1) into B6 recipients (Thy1.2) followed by infection with either Vir LM-GP_33_ (1 × 10^4^ CFU i.v.) or LCMV (2 × 10^5^ PFU i.p.). 2° infections with Att LM (5 × 10^6^ CFU/mouse) or Vir LM (1 × 10^4^, 1 × 10^5^, or 1 × 10^6^ CFU/mouse) either expressing or not expressing GP_33_ were administered i.v. >30 days after 1° infection. For adoptive transfer of 1° memory cells, splenocytes from mice infected >30 days previously with LCMV were stained with PE-anti Thy1.1 antibodies and purified with anti-PE magnetic bead sorting using standard AutoMacs protocols. B6 or GKO mice received adoptive transfer of 400,000 1° memory P14 cells i.v. As a measure of protection, body weight was monitored daily, and CFUs were determined in the spleens and liver at the indicated times post 2° infection^38^. Ampicillin treated mice received i.p. injection (2mg in H_2_O) and drinking water (2 mg/ml) for 24 hrs^39^.

### Detection of Memory Cells and *ex vivo* P14 Cell Analysis

Total memory CD8 T cells were detected using the surrogate activation markers CD8 and CD11a as previously described^40^. Endogenous GP_33_-specific memory CD8 T cells were detected with MHC class I GP_33_ tetramers, and memory P14 cells were gated on CD8/Thy1.1+ cells. *Ex vivo* activation status following infection was determined by incubating splenocytes at 37 °C for 1 h in the presence of BrefeldinA (BFA) followed by intracellular staining with antibodies (Abs) against either IFN-γ or GrB or extracellular staining with Abs against either CD25 or CD69. All cells for flow cytometry were analyzed using a FACSCanto flow cytometer and analyzed using FlowJo software.

### *In vitro* Activation of P14 Cells

Splenocytes were isolated from mice containing memory P14 cells generated following LCMV infection and were incubated for 4 hrs in the presence of media, GP_33_ peptide (0.01 or 200 nM), rIL-12 and IL-18 or IL-12 and TNF-α or IL-18 and IFN-β (0.5 ng/mL each), or 0.01 nM GP_33_ peptide and 0.5 ng/mL each of recombinant cytokine combinations. Cells were incubated for 1 additional hour in the presence of BFA before being stained extracellularly with Abs against CD25 or CD69, or intracellularly with Abs against IFN-γ.

### Detection of Serum IL-12

Blood samples were collected from mice 4, 8, and 24 hrs post 2° infection and from mice that did not receive 2° infection, and serum was collected after centrifugation. IL-12 was measured using a mouse IL-12 platinum ELISA (eBioscience), and absorbance values (450 nm) were measured and assessed using Gen5 software (BioTek).

### Statistical Analysis

Statistical analyses were performed using GraphPad Prism software version 6 (GraphPad Sofware Inc., San Diego, CA). Statistical comparisons of two groups were done using the Mann-Whitney test and the unpaired t test. Statistical comparisons of more than two groups was done using one-way ANOVA with the Bonferroni posttest. R squared values were calculated from linear regression analysis.

## Additional Information

**How to cite this article**: Martin, M. D. and Badovinac, V. P. Antigen-dependent and -independent contributions to primary memory CD8 T cell activation and protection following infection. *Sci. Rep.* **5**, 18022; doi: 10.1038/srep18022 (2015).

## Supplementary Material

Supplementary Information
