# Supplementary material for: Antigen-dependent and –independent contributions to primary memory CD8 T cell activation and protection following infection
Source: Sci Rep. 2015 Dec 10;5:18022. doi: 10.1038/srep18022 (PMC4675085; doi:10.1038/srep18022)
Supplement: Supplementary Information [file srep18022-s1.pdf]

Antigen-dependent and –independent contributions to primary memory CD8 T cell activation and protection following infection

Matthew D. Martin and Vladimir P. Badovinac

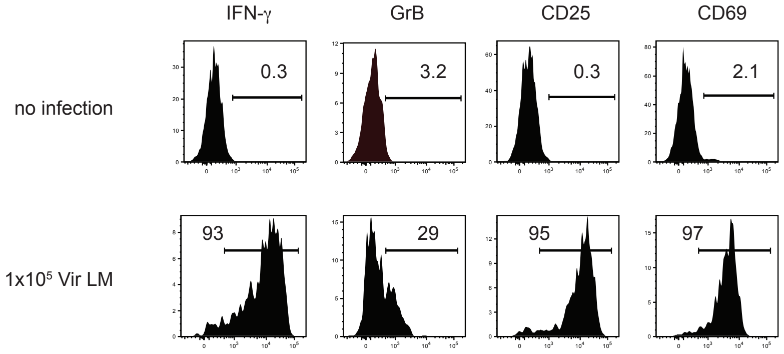

Supplemental Figure 1. P14 responses are driven by 2º infection. Memory P14 cells were generated by transferring naive P14 cells into mice followed by LCMV infection. Mice either received 2º infection with $1x10^5$ CFU Vir LM or did not receive secondary infection. Representative histograms of IFN-γ, GrB, CD25, and CD69 expression on splenocytes.
